# Viabilidade e Segurança de Alta Hospitalar Precoce após TAVI com Abordagem Minimalista no SUS

**DOI:** 10.36660/abc.20230328

**Published:** 2023-10-03

**Authors:** Marcos Almeida Meniconi, Fernanda Jacques Calçado Oliveira, Alberto Colella Cervone, Dorival Julio Della Togna, Fausto Feres, Auristela Isabel de Oliveira Ramos, Dimytri Alexandre de Alvim Siqueira

**Affiliations:** 1 Hospital das Clínicas Faculdade de Medicina Universidade de São Paulo São Paulo SP Brasil Instituto do Coração do Hospital das Clínicas da Faculdade de Medicina da Universidade de São Paulo, São Paulo, SP – Brasil; 2 Instituto Dante Pazzanese de Cardiologia São Paulo SP Brasil Instituto Dante Pazzanese de Cardiologia, São Paulo, SP – Brasil

**Keywords:** Substituição da Valva Aórtica Transcateter, Estenose da Valva Aórtica, Sistema Único de Saúde

## Abstract

**Figure:**
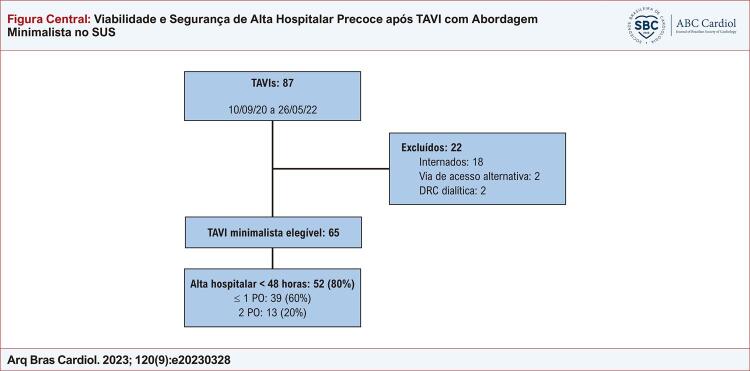
: Viabilidade e Segurança de Alta Hospitalar Precoce após TAVI com Abordagem Minimalista no SUS

**Figure:** : Viabilidade e Segurança de Alta Hospitalar Precoce após TAVI com Abordagem Minimalista no SUS

Resultados após implante de prótese aórtica por cateter por abordagem minimalista. DRC: doença renal crônica; PO: dias pós-operativos; TAVI: implante por cateter de prótese aórtica.

## Introdução

O implante por cateter de prótese aórtica (TAVI, do inglês *transcatheter aortic valve implantation*) estabeleceu-se como tratamento de escolha para o tratamento de octogenários portadores de estenose aórtica.^1^ Abrangentemente, denomina-se TAVI com abordagem minimalista (TAVI-M) a realização do procedimento sob sedação consciente e anestesia local, acesso femoral percutâneo, monitorização com ecocardiograma transtorácico e mobilização precoce. Estudos apontam para a segurança do TAVI-M com alta hospitalar em até 24 horas após o procedimento^2,3^ e a redução de custos hospitalares^4,5^– aspecto relevante sob a perspectiva da saúde pública nacional. Desta forma, esta pesquisa tem como objetivo avaliar a exequibilidade e a segurança de um protocolo multidisciplinar institucional de TAVI minimalista visando alta hospitalar em até 48 horas, implementado em hospital terciário do Sistema Único de Saúde (SUS).

## Métodos

Estudo observacional, prospectivo e unicêntrico, com seleção de pacientes consecutivamente submetidos a TAVI no período de Setembro de 2020 a Maio de 2022.

### Critérios de inclusão e exclusão

Foram selecionados pacientes ≥ 18 anos, portadores de estenose aórtica importante com indicação eletiva de TAVI. Foram excluídos: portadores de disfunção ventricular esquerda importante (fração de ejeção do ventrículo esquerdo < 30%); necessidade via de acesso alternativa (não femoral); clearance de creatinina < 15 ml/min/1,73 m^2^; presença de discrasia sanguínea ou plaquetopenia severa (< 50.000/mm^3^); realização de outro procedimento cirúrgico ou intervencionista percutâneo na mesma internação.

### Coleta de dados e análise estatística

Os dados foram coletados através de questionário, prontuário eletrônico e/ou contato telefônico, realizados sistematicamente 30 dias após a alta hospitalar. As variáveis quantitativas foram apresentadas como média ± desvio padrão ou variação interquartil. As variáveis categóricas foram expressas como uma proporção do todo (%).

Os desfechos clínicos analisados compreenderam óbito por todas as causas, óbito por causas cardiovasculares, acidente vascular cerebral, complicações vasculares e hemorrágicas e necessidade de marca-passo definitivo em 30 dias; avaliou-se ainda o tempo de internação e a necessidade de readmissões hospitalares em 30 dias. Os desfechos foram definidos de acordo com o Valve Academic Research Consortium 3.^6^

## Resultados

De Setembro de 2020 a Maio de 2022, 87 pacientes submeteram-se ao TAVI, sendo 65 pacientes (74,7%) submetidos à estratégia minimalista; 22 apresentavam critérios de exclusão (Figura Central). Não houve perda de seguimento.

Os pacientes submetidos a TAVI-M apresentavam média de idade de 79,9 ± 4,8 anos, sendo 27 (41,5%) mulheres; baixo risco cirúrgico, conforme escores STS e Euroscore II (média de 2,4% ± 1,45% e 3,0% ± 2,15%, respectivamente). As comorbidades mais prevalentes foram: hipertensão arterial sistêmica em 51 (78,4%) indivíduos, diabetes mellitus em 26 (40%) e doença arterial coronariana em 25 (38,4%). Seis pacientes (9,2%) possuíam valva aórtica bicúspide (Tabela 1).

**Tabela 1:** – Características basais

Características	n (desvio padrão)
Idade (anos)	79,9 (± 4,8)
Sexo feminino	27 (41,5%)
Índice de massa corporal (kg/m^2^)	28,4 (± 6,2)
Distância até domicílio (km)	19,4 (± 7,5)*
Classe funcional NYHA	I: 4 (6,2%)
	II: 40 (61,5%)
	III: 21 (32,3%)
	IV: 0
Grau de angina CCS	Sem angina: 43 (66%)
	I: 1 (1,5%)
	II: 15 (23%)
	III: 5 (8%)
	IV: 1 (1,5%)
Hipertensão arterial sistêmica	51 (78,4%)
Diabetes mellitus	26 (40%)
Dislipidemia	35 (53,8%)
Clearance de creatinina (mL/min/1,73 m^2^)	> 60: 37 (57%)
	45 a 59: 19 (29,2%)
	30 a 44: 9 (13,8%)
Doença arterial coronariana	25 (38,4%)
Acidente vascular encefálico prévio	6 (9,2%)
Neoplasia	9 (13,8%)
Tabagismo	21 (32,3%)
Doença pulmonar obstrutiva crônica	3 (4,6%)
Doença arterial periférica	7 (10,8%)
Cirurgia de revascularização prévia	10 (15,4%)
Intervenção coronária percutânea	5 (7,7%)
Válvula aórtica bicúspide	6 (9,2%)
STS score (%)	2,4 (± 1,45)
Euroscore II (%)	3,0 (± 2,15)

* Excluíram-se outliers (valores > 53,1 km e < 6,9 km).

O eletrocardiograma à admissão revelou: ritmo sinusal em 52 (80%) indivíduos; fibrilação atrial, 9 (13,8%); ritmo de marca-passo definitivo, 4 (6,2%); bloqueio atrioventricular de primeiro grau, 14 (21,5%); bloqueio de ramo esquerdo, 7 (10,8%); e bloqueio de ramo direito, 5 (7,7%).

As próteses utilizadas, quantidade de contraste e realização de pré- ou pós-dilatação estão descritas na Tabela 2.

**Tabela 2:** – Características do procedimento

Tipo de prótese	Evolut R^®^ (Medtronic^®^): 1 (1,5%) Sapien 3^®^ (Edwards Lifesciences^®^): 14 (21%) Accurate Neo^®^ (Boston Scientific^®^): 12 (18,5%) Myval^®^ (Meril Life Sciences^®^): 38 (59%)
Pré-dilatação (n)	34 (52,3%)
Pós-dilatação (n)	19 (29,2%)
Contraste (ml)	108 (± 30)

O ecocardiograma transtorácico de base demonstrou: área valvar aórtica média de 0,65 (± 0,15) cm^2^, gradiente transvalvar aórtico médio de 53 ± 18,4 mmHg, pressão sistólica da artéria pulmonar média estimada de 35 ± 12 mmHg e fração de ejeção do ventrículo esquerdo média de 55,2% ± 0,11% (método de Simpson). Imediatamente após o TAVI, a média da área de orifício efetivo aórtico elevou-se para de 2,1 ± 0,5 cm^2^ e o gradiente transvalvar aórtico médio foi de 4,7 (± 3,5) mmHg. A incidência de *leak* paravalvar ≥ moderado foi de 4,6% (n = 3).

Ocorreram 2 óbitos: 1 de causa cardiovascular e outro por infecção pelo vírus SARS-CoV-2. Dois pacientes (3%) requereram o implante de marca-passo definitivo. Sangramentos maiores ocorreram em 3 (4,6%) casos, com necessidade de conversão cirúrgica (Tabela 3).

**Tabela 3:** – Desfechos clínicos intra-hospitalares

Desfechos intra-hospitalares	n (%)
Óbito total	2 (3%)
Óbito de causa cardiovascular	1 (1,5%)
Acidente vascular encefálico	0
Complicação vascular maior	1 (1,5%)
Sangramento maior e ameaçador à vida	3 (4,6%)
Implante de marca-passo definitivo	2 (3%)
Conversão anestésica	3 (4,6%)
Rotura de anel valvar	1 (1,5%)
Obstrução coronária	0
Implante de segunda prótese	0

### Tempo de internação

**Figura 1:**
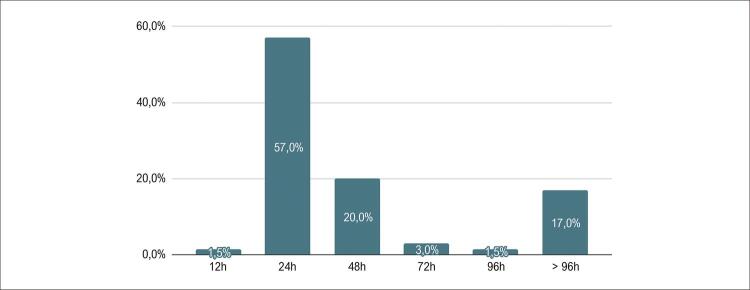
– Tempo para alta hospitalar após o TAVI-M.

O tempo médio de internação foi de 52,1 horas ou 2,17 dias. Alta hospitalar em menos de 48 horas pós-TAVI foi concretizada em 52 (80%) pacientes, sendo que 39 (60%) receberam alta em até 24 horas (Figura 1).

A alta hospitalar foi postergada (> 48 horas) devido a presença de *leak* paravalvar moderado (n = 1), lesão renal aguda pós-renal (n = 1), pseudoaneurisma de artéria femoral (n = 1), complicação vascular maior (n = 1), sangramento menor (n = 1), conversão cirúrgica (n = 1) e distúrbios de condução (n = 5).

### Readmissões hospitalares

Quatro pacientes (6,1%) reinternaram em 30 dias, sendo dois (3%) por causas cardiovasculares (insuficiência cardíaca descompensada e acidente vascular encefálico). As causas não cardiovasculares foram fratura patológica de fêmur e epistaxe. Nenhum óbito ocorreu após a alta hospitalar em 30 dias.

## Discussão

O tempo médio de internação de 2,17 dias é semelhante ao do grande registro estadunidense de 2019^7^ e ensaio clínico 3M-TAVR.^3^ Nesta série, a indicação de marca-passo definitivo em 30 dias foi de apenas 3%, em contraponto aos 20,1% do registro nacional multicêntrico de 2008 a 2015.^8^ Este resultado que pode ser explicado pelo predomínio de próteses balão-expansíveis e implante mais alto em relação ao anel valvar.

### Limitações

O trabalho de único centro pode limitar a reprodutibilidade e a generalização dos resultados do protocolo instituído. O reduzido número de pacientes nesta amostra impede análise estatística de maior robustez. Este estudo também carece de uma análise de custo-efetividade, assunto de interesse para novas iniciativas.

## Conclusão

Nesta experiência inicial, a aplicação de um protocolo institucional de TAVI-M mostrou-se segura e exequível em hospital do SUS, refletindo-se em resultados clínicos satisfatórios, reduzido tempo de internação e baixas taxas de readmissão hospitalar.
