# Supplementary material for: Viabilidade e Segurança de Alta Hospitalar Precoce após TAVI com Abordagem Minimalista no SUS
Source: Arq Bras Cardiol. 2023 Oct 3;120(9):e20230328. [Article in Portuguese] doi: 10.36660/abc.20230328 (PMC10547432; doi:10.36660/abc.20230328)
Supplement: Translation

# Translation: Viability and Safety of Early Hospital Discharge after Minimalist TAVI in the Brazilian Unified Health System

**Authors:** Marcos Almeida Meniconi, Fernanda Jacques Calçado Oliveira, Alberto Colella Cervone, Dorival Julio Della Togna, Fausto Feres, Auristela Isabel de Oliveira Ramos, Dimytri Alexandre de Alvim Siqueira

**Affiliations:** 1 Brazil Instituto do Coração do Hospital das Clínicas da Faculdade de Medicina da Universidade de São Paulo, São Paulo, SP – Brazil; 2 Brazil Instituto Dante Pazzanese de Cardiologia, São Paulo, SP – Brazil

**Keywords:** Transcatheter Aortic Valve Replacement, Aortic Valve Stenosis, Unified Health System

## Abstract

**Figure:** : Viability and Safety of Early Hospital Discharge after Minimalist TAVI in the Brazilian Unified Health System

## Introduction

Transcatheter aortic valve implantation (TAVI) has established itself as the treatment of choice for octogenarian patients with aortic stenosis.^1^ Broadly speaking, TAVI with a minimalist approach (M-TAVI) refers to the performance of the procedure under conscious sedation and local anesthesia, percutaneous femoral access, monitoring with transthoracic echocardiography, and early mobilization. Studies have indicated the safety of M-TAVI with hospital discharge within 24 hours after the procedure^2,3^ with reduced hospital costs,^4,5^which is a relevant aspect from the perspective of national public health. Thus, the objective of this study was to evaluate the feasibility and safety of a multidisciplinary institutional M-TAVI protocol with the goal of hospital discharge within 48 hours, implemented in a tertiary hospital that is part of the Brazilian Unified Health System (SUS).

## Methods

This was an observational, prospective, single-center study with a selection of patients who consecutively underwent TAVI from September 2020 to May 2022.

### Inclusion and exclusion criteria

Patients aged ≥ 18 years, with important aortic stenosis and elective indication for TAVI were selected. Patients with any of the following were excluded: significant left ventricular dysfunction (left ventricular ejection fraction < 30%); need for alternative access route (other than femoral); creatinine clearance < 15 ml/min/1.73 m^2^; presence of blood dyscrasia or severe thrombocytopenia (< 50,000/mm^3^); performance of another surgical or percutaneous interventional procedure during the same hospitalization period.

### Data collection and statistical analysis

Data were collected by means of a questionnaire, electronic medical records, and/or telephone contact, systematically applied 30 days after hospital discharge. Quantitative variables were presented as mean ± standard deviation or interquartile range. Categorical variables were expressed as a proportion of the whole (%).

The clinical outcomes analyzed included death from all causes, death from cardiovascular causes, stroke, vascular and hemorrhagic complications, and the need for a permanent pacemaker within 30 days; the length of hospital stay and the need for hospital readmissions within 30 days were also evaluated. Outcomes were defined according to the Valve Academic Research Consortium 3.^6^

## Results

From September 2020 to May 2022, 87 patients underwent TAVI, with 65 patients (74.7%) undergoing the minimalist strategy; 22 met exclusion criteria (Central Figure). There was no loss to follow-up.

Patients who underwent M-TAVI had a mean age of 79.9 ± 4.8 years, and 27 (41.5%) were women. Patients had low surgical risk, according to the STS and Euroscore II scores (means of 2.4% ± 1.45% and 3.0% ± 2.15%, respectively). The most prevalent comorbidities were systemic arterial hypertension in 51 (78.4%) patients, diabetes mellitus in 26 (40%), and coronary artery disease in 25 (38.4%). Six patients (9.2%) had a bicuspid aortic valve (Table 1).

**Table 1:** – Baseline characteristics

Characteristics	n (standard deviation)
Age (years)	79.9 (± 4.8)
Female sex	27 (41.5%)
Body mass index (kg/m^2^)	28.4 (± 6.2)
Distance to home (km)	19.4 (± 7.5)*
NYHA functional class	I: 4 (6.2%)
	II: 40 (61.5%)
	III: 21 (32.3%)
	IV: 0
CCS angina grade	Sem angina: 43 (66%)
	I: 1 (1.5%)
	II: 15 (23%)
	III: 5 (8%)
	IV: 1 (1.5%)
Systemic arterial hypertension	51 (78.4%)
Diabetes mellitus	26 (40%)
Dyslipidemia	35 (53.8%)
Creatinine clearance (mL/min/1.73 m^2^)	> 60: 37 (57%)
	45 a 59: 19 (29.2%)
	30 a 44: 9 (13.8%)
Coronary artery disease	25 (38.4%)
Prior stroke	6 (9.2%)
Neoplasia	9 (13.8%)
Tobacco use	21 (32.3%)
Chronic obstructive pulmonary disease	3 (4.6%)
Peripheral arterial disease	7 (10.8%)
Prior revascularization surgery	10 (15.4%)
Percutaneous coronary intervention	5 (7.7%)
Bicuspid aortic valve	6 (9.2%)
STS score (%)	2.4 (± 1.45)
Euroscore II (%)	3.0 (± 2.15)

* Outliers were excluded (values > 53.1 km and < 6.9 km).

Admission electrocardiography revealed the following: sinus rhythm in 52 (80%) patients, atrial fibrillation in 9 (13.8%), permanent pacemaker rhythm in 4 (6.2%), first-degree atrioventricular block in 14 (21.5%), left bundle branch block in 7 (10.8%), and right bundle branch block in 5 (7.7%).

Table 2 describes the prostheses used, amount of contrast, and performance of pre- or post-dilation.

**Table 2:** – Procedure characteristics

Prosthesis type	Evolut R^®^ (Medtronic^®^): 1 (1.5%) Sapien 3^®^ (Edwards Lifesciences^®^): 14 (21%) Accurate Neo^®^ (Boston Scientific^®^): 12 (18.5%) Myval^®^ (Meril Life Sciences^®^): 38 (59%)
Pre-dilation (n)	34 (52.3%)
Post-dilation (n)	19 (29.2%)
Contrast (ml)	108 (± 30)

Baseline transthoracic echocardiogram demonstrated the following: mean aortic valve area of 0.65 (± 0.15) cm^2^, mean aortic transvalvular gradient of 53 ± 18.4 mmHg, estimated mean pulmonary artery systolic pressure of 35 ± 12 mmHg, and mean left ventricular ejection fraction of 55.2% ± 0.11% (Simpson method). Immediately after TAVI, the mean effective aortic orifice area increased to 2.1 ± 0.5 cm^2^, and the mean aortic transvalvular gradient was 4.7 (± 3.5) mmHg. The incidence of paravalvular leak ≥ moderate was 4.6% (n = 3).

Two deaths occurred: 1 due to cardiovascular causes and 1 due to SARS-CoV-2 infection. Two patients (3%) required permanent pacemaker implantation. Major bleeding occurred in 3 (4.6%) cases, requiring surgical conversion (Table 3).

**Table 3:** – In-hospital clinical outcomes

In-hospital outcomes	n (%)
Total deaths	2 (3%)
Death from cardiovascular causes	1 (1.5%)
Stroke	0
Major vascular complication	1 (1.5%)
Major life-threatening bleeding	3 (4.6%)
Permanent pacemaker implantation	2 (3%)
Anesthetic conversion	3 (4.6%)
Valve annulus rupture	1 (1.5%)
Coronary obstruction	0
Second prosthesis implant	0

### Length of hospital stay

The mean length of hospital stay was 52.1 hours or 2.17 days. Hospital discharge in under 48 hours after TAVI was achieved in 52 (80%) patients, with 39 (60%) discharged within 24 hours (Figure 1).

**Figure 1:** – Time to hospital discharge after M-TAVI.

Hospital discharge was delayed (> 48 hours) due to presence of moderate paravalvular leak (n = 1), post-renal acute kidney injury (n = 1), femoral artery pseudoaneurysm (n = 1), major vascular complication (n = 1), minor bleeding (n = 1), surgical conversion (n = 1), and conduction disorders (n = 5).

### Hospital readmissions

Four patients (6.1%) were readmitted within 30 days, 2 of them (3%) due to cardiovascular causes (decompensated heart failure and stroke). The non-cardiovascular causes were pathological fracture of the femur and epistaxis. No deaths occurred within 30 days after hospital discharge.

## Discussion

The mean length of hospital stay of 2.17 days is similar to that of a large 2019 registry from the United States^7^ and the 3M-TAVR clinical trial.^3^ In this series, the indication for permanent pacemaker within 30 days was only 3%, in contrast to 20.1% in the Brazilian national multicenter registry from 2008 to 2015.^8^ This result can be explained by the predominance of balloon-expandable prosthesis and higher implant in relation to the valve annulus.

### Limitations

The single-center design of this study can limit the reproducibility and generalization of the results of the protocol instituted. The small number of patients in this sample prevents a more robust statistical analysis. This study also lacks cost-effectiveness analysis, which is a subject of interest for future initiatives.

## Conclusion

In this initial experience, the application of an institutional M-TAVI protocol proved to be safe and feasible in a SUS hospital, as reflected by satisfactory clinical results, reduced hospitalization time, and low hospital readmission rates.
